# The Roles of Transient Receptor Potential Vanilloid 1 and 4 in Pneumococcal Nasal Colonization and Subsequent Development of Invasive Disease

**DOI:** 10.3389/fimmu.2021.732029

**Published:** 2021-11-03

**Authors:** Masamitsu Kono, Denisa Nanushaj, Hideki Sakatani, Daichi Murakami, Masayoshi Hijiya, Tetsuya Kinoshita, Tatsuya Shiga, Fumie Kaneko, Keisuke Enomoto, Gen Sugita, Masayasu Miyajima, Yuka Okada, Shizuya Saika, Muneki Hotomi

**Affiliations:** ^1^ Department of Otorhinolaryngology-Head and Neck Surgery, Wakayama Medical University, Wakayama, Japan; ^2^ Department of Ophthalmology, Wakayama Medical University, Wakayama, Japan

**Keywords:** transient receptor potential (TRP), *Streptococcus pneumoniae* (pneumococcus), nasal colonization, pneumonia, sepsis, mouse model, influenza virus

## Abstract

Transient receptor potential (TRP) channels, neuronal stimulations widely known to be associated with thermal responses, pain induction, and osmoregulation, have been shown in recent studies to have underlying mechanisms associated with inflammatory responses. The role of TRP channels on inflammatory milieu during bacterial infections has been widely demonstrated. It may vary among types of channels/pathogens, however, and it is not known how TRP channels function during pneumococcal infections. *Streptococcus pneumoniae* can cause severe infections such as pneumonia, bacteremia, and meningitis, with systemic inflammatory responses. This study examines the role of TRP channels (TRPV1 and TRPV4) for pneumococcal nasal colonization and subsequent development of invasive pneumococcal disease in a mouse model. Both TRPV1 and TRPV4 channels were shown to be related to regulation of the development of pneumococcal diseases. In particular, the influx of neutrophils (polymorphonuclear cells) in the nasal cavity and the bactericidal activity were significantly suppressed among TRPV4 knockout mice. This may lead to severe pneumococcal pneumonia, resulting in dissemination of the bacteria to various organs and causing high mortality during influenza virus coinfection. Regulating host immune responses by TRP channels could be a novel strategy against pathogenic microorganisms causing strong local/systemic inflammation.

## Introduction

Temperature is one of the most critical factors for organisms to maintain their lives. For maintenance of a suitable environment for survival, there are various temperature sensing mechanisms in many organisms. An environment of too high or low temperature is a life-threatening condition that is recognized as a sense of pain. The transient receptor potential vanilloid (TPRV) 1 channel was firstly identified in 1997 as one of the main molecules involved in temperature sensing systems (1). Transient receptor potential (TRP) channels are activated not only by temperature but also by multiple external stimuli such as taste, acidity, or pain and have a critical role in maintaining homeostasis. TRP channels have been shown in recent studies to be associated with various diseases, including infection by microorganisms (2–6).

TRPV channels are evolutionarily conserved and important in the signaling detection; they are activated by vanilloid-like compounds (7) and mostly expressed on the glial cells: microglia and astrocytes (8). However, some of the channels, such as TRPV1 and TRPV4, are found to be expressed in the upper respiratory tract and lungs. TRPV1 is a capsaicin-activated channel found in the peripheral nerves that is responsible for the burning pain in cases of inflammation (9), which is thought to have a strong relation with respiratory tract inflammatory diseases, asthma, and chronic obstructive pulmonary disease (10, 11). On the other hand, TRPV4 has been found to be activated by lipopolysaccharide (LPS), which is the main virulence factor of Gram-negative bacteria and a strong agonist of toll-like receptor 4 (3). TRPV4 is reported to be associated with the pathogenesis of chronic rhinosinusitis (12). The upper respiratory tract is the site where commensal bacterial flora is formed early in life. These bacteria sometimes invade deeper tissues or migrate to the other sites that cause local inflammation and tissue damage. TRP channels are thought to have potentially critical roles in the defense system by repairing the tissue in the upper respiratory tract damaged by infection, but the association between TRP and bacterial infections present in the upper respiratory tract is not well understood.

*Streptococcus pneumoniae*, a Gram-positive bacterium that does not have LPS, is a representative commensal of the nasopharynx that also can become pathogenic and cause diseases, such as otitis media, bacteremia, pneumonia, and meningitis in the cases of migration to the more sterile sites (13). It is also associated with potentially lethal secondary bacterial infection during viral infections, such as influenza virus (14). Despite the introduction of pneumococcal conjugate vaccines, there are still many outstanding problems to be resolved: the high evolution rate, serotype replacement, and the high risk of mortality by invasive pneumococcal infection within the immunologically immature population (15, 16). Our recent study revealed the invasion of *S. pneumoniae* into the cell-to-cell junction and the formation of clusters beneath the layer of human epithelial cells, suggesting the mechanism of pneumococcal colonization and invasion to deeper tissue (17). In this study, we hypothesized that TRPV channels have protective functions for the host by regulating inflammation and tissue damage in the course of pneumococcal nasal colonization and subsequent development of severe invasive infections. We evaluated the role of TRPV1 and TRPV4 channels on pneumococcal pathogenesis using a nasal colonization mouse model.

## Materials and Methods

### Ethics Statement

This study was conducted according to the guidelines outlined by the National Science Foundation Animal Welfare Requirements and the Wakayama Medical University Animal Care and Use Committee. The study was approved by the Institutional Animal Care and Use Committee at Wakayama Medical University (Approval number: 901).

### Bacterial Strain and Growth Conditions

A pneumococcal strain, P2431 (clinical isolate of serotype 6A), which can establish stable colonization and cause sepsis, was used in this study (18). The bacteria were grown in Todd–Hewitt broth containing 0.5% yeast extract (THY) at 37°C until the mid-log phase and stocked in aliquots at known CFU concentrations in THY broth containing 10% glycerol at –80°C until the experiment.

### Mice

The mice used in this experiment were around 6 weeks old. C57BL/6J wild-type mice were purchased from Charles River Laboratories Japan, Inc. (Yokohama, Japan). TRPV1 KO mice were purchased from the Jackson Laboratory (Bar Harbor, ME, USA), and TRPV4 KO mice were introduced from RIKEN BRC (Tsukuba, Japan) (19, 20). All mice were maintained in a conventional animal facility at Wakayama Medical University.

### Infection

**Figure 1:**
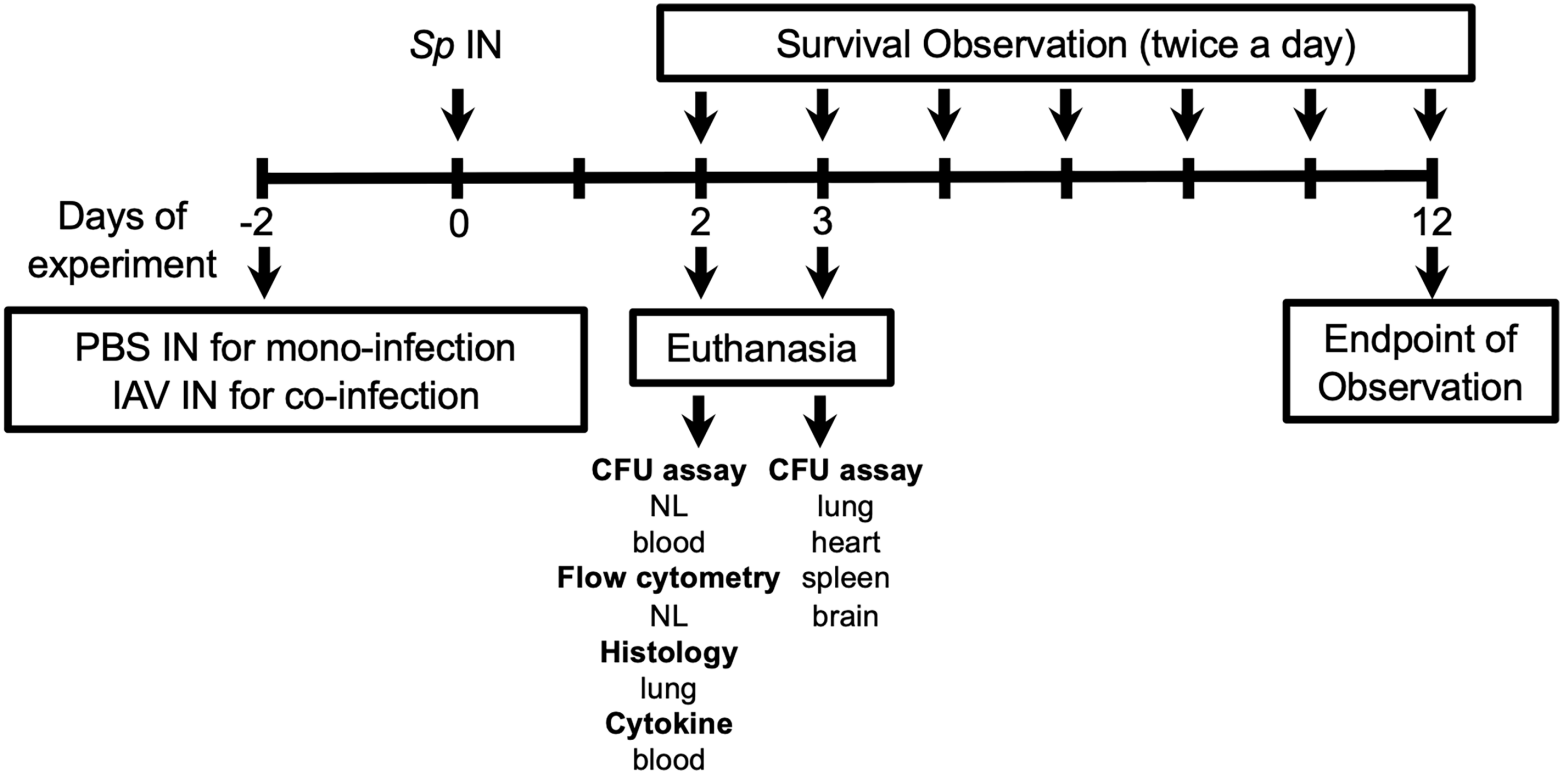
Experimental schematic for this study. Mice were intranasally (IN) inoculated with PBS or IAV on day –2, and *S. pneumoniae* (*Sp*) on day 0. On day 2, nasal lavage (NL) for pneumococcal colony counts (CFU assay) and flow cytometry, lung for histology, blood for CFU assay, and cytokine quantification (ELISA) were collected. On day 3, lung, heart, spleen, and brain were collected for CFU assay. For evaluating the development of lethal infection, mice were monitored until they displayed signs of sick and confirmed bacteremia. NL, nasal lavage.

The schematic of the experiments is shown in **Figure 1**. This study was designed to investigate a role of TRPV channels in the course of the development of invasive pneumococcal infections following nasal colonization. For the pneumococcal mono-infection group, the mice were intranasally administered with 10 µl of phosphate-buffered saline (PBS) on day –2, and with *S. pneumoniae* on day 0. For the influenza A virus (IAV) coinfection group, the mice were intranasally administered with 10 µl of IAV (HKx31, H3N2 strain) (2 × 10^3^ TCID_50_) on day –2, and with *S. pneumoniae* on day 0. To avoid the mice aspirating the bacteria to the lower respiratory tract and causing pneumonia directly, the mice were inoculated to colonize *S. pneumoniae* intranasally without anesthesia, as previously reported (21–23). The mice were sacrificed by isoflurane asphyxiation for sampling on the desired experimental day or monitored until the mice showed signs of lethal infection including sepsis and sustained bacteremia (decreased motor activity, shivering, and messy hair).

The upper respiratory tract was lavaged with 200 µl of sterile PBS from a needle (26 gauge) inserted into the trachea, and the fluid was collected in a sterile tube. Twenty microliters of the lavage was serially diluted and incubated on a blood plate overnight so the colonies could be counted. Approximately 500 µl of blood was collected by cardiac puncture from each mouse. After taking 20 µl for colony counting, 480 µl of the blood was centrifuged and the serum was stocked for ELISA. The lungs, spleens, hearts, and brains were obtained after the removal of the blood by cardiac puncture. After washing thoroughly with sterile PBS, the organs were homogenized in 1 ml of PBS for counting colonies or were put in 4% of paraformaldehyde (PFA) for histology.

### Histological Analyses

The spleens and the lungs were placed on the tubes consisting of 1 ml PFA. The organs were then placed in the automatic sample processor and dehydrated for 48 h. After the cooling process, the tissues were each cut 5 mm thick by microtome and were stored until hematoxylin and eosin (H & E) staining analysis.

The paraffin was fixed for 20 min in the incubator (67°C). The slides were cooled at room temperature for 15 min. The sections were deparaffinized with lemosol two times for 5 min each. The sections were then treated in the following order: 100% ethanol for 1 min × 2, 95% ethanol for 1 min × 2, 80% ethanol for 1 min, 70% ethanol for 1 min. The slides were soaked in Mayer’s hematoxylin solution for 7 min and then rinsed in tap water. The slides were soaked in an eosin alcohol solution for 1.5 min and then rinsed in tap water. The tissues were dehydrated in the following order for 1 min each: 70%, 80%, 95%, 95%, 100%, and 100% ethanol). The tissues were cleaned in lemosol for 1 min two times. Then, the slides were coverslipped with Canada balsam mounting solution.

The severity of lung inflammation was quantified using the scoring system proposed in a previous report (24). In brief, the lung tissue was evaluated by five independent factors (neutrophils in the alveolar space, neutrophils in the interstitial space, hyaline membranes, proteinaceous debris filling the air spaces, alveolar septal thickening) indicating the degree of inflammation and calculated using the original formula (**Supplementary Table**). Six samples were evaluated for each group.

### Flow Cytometry

Nasal lavages were pelleted at 1,500 rpm for 2 min and resuspended in 200 µl PBS containing 1% bovine serum albumin (BSA). After FcR blocking with a 1:200 dilution of anti-CD16/32 (BioLegend, San Diego, CA, USA) for 15 min, cells were stained for 30 min at 4°C with 25 µl of 1:150 dilution of the following antibodies: anti-CD11b-V450 (BD Biosciences, San Jose, CA, USA), anti-F4/80-PE (BioLegend), anti-Ly6G-PerCP-Cy (BD Biosciences), and anti-CD45-APC-Cy7 (BD Biosciences) for 30 min on ice in the dark. Cells were washed with PBS with 1% BSA, then fixed with 4% PFA. FACSVerse (BD) was used for flow cytometry analysis. After excluding dead cells and debris by gating with forward and side scatter, neutrophils (polymorphonuclear cells; PMNs) were detected as CD11b^+^, Ly6G^+^, and CD45^+^ components. Macrophages were detected as F4/80^+^, Ly6G^-^, and CD45^+^ components. The absolute number of cells in each sample was counted.

### Enzyme-Linked Immunosorbent Assay (ELISA)

The blood samples were centrifuged at 5,000 rpm for 5 min and the sera were collected for enzyme-linked immunosorbent assay (ELISA). Quantitative evaluation of cytokines (TNF-α, IL-6, and IL-1β) in serum was performed by following the ELISA kit protocol (Proteintech, Rosemont, IL, USA).

### Bactericidal Assay

To assess the impact of TRPV1 and TRPV4 on the neutrophil phagocytic function, we performed bactericidal assays using murine PMNs ex vivo as previously reported (25). Bone marrow cells were flushed from the 6-week-old murine femora and tibia with RPMI 1640 (Thermo Fisher Scientific, Waltham, MA, USA) with 10% fetal bovine serum and 2 mM EDTA. By centrifugation for 30 min at 2,000 rpm, PMNs were isolated with a discontinuous gradient of Histopaque-1077 and -1119 (Sigma-Aldrich, St. Louis, MO, USA). The cells were then resuspended with Hank’s Balanced Salt Solution (Thermo Fisher Scientific) and adjusted to the desired concentration. We confirmed that more than 90% of the CD45^+^ cells isolated were Ly6G^+^ and CD11b^+^. The bacteria (10^2^ CFU) were pre-opsonized with baby rabbit complement (Pel-Freez Biologicals, Rogers, AR, USA) for 30 min and applied to the PMNs in a bacterium: PMN ratio of 1:1,000. After 45 min of incubation at 37°C, the bacteria were incubated on the blood agar overnight and the number of colonies counted for quantification. The bacterial survival rate was calculated by dividing the number of bacteria in the experimental sample by those in the control that did not contain PMNs.

### Statistical Analyses

All analyses were performed by GraphPad Prism 8.4.3 (GraphPad Software, San Diego, CA, USA). Analysis of the survival experiment was performed by the Kaplan–Meier log-rank test. The Kruskal–Wallis test with Dunn’s multiple-comparison test was applied to compare the three groups. *p* < 0.05 was considered to indicate a significant difference.

## Results

### Nasal Colonization and Development of Bacteremia After Intranasal Inoculation With a Virulent Strain 6A

**Figure 2:**
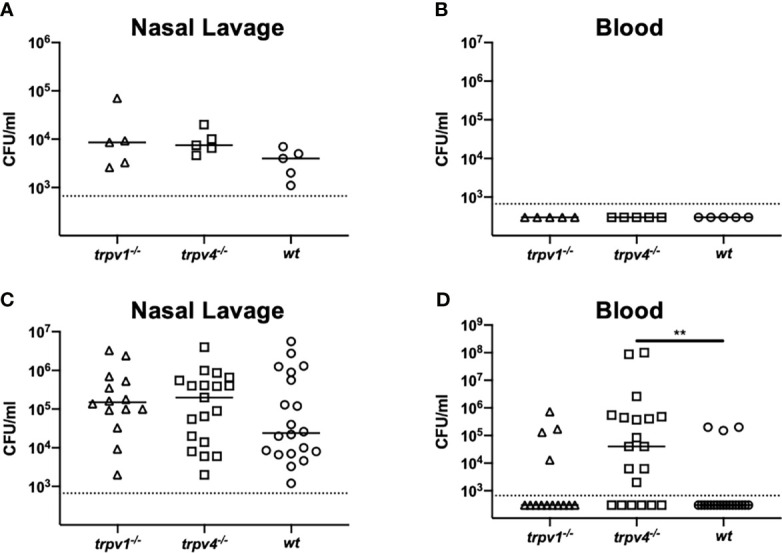
Nasal colonization and bacteremia in the mono-infection model. Mice were intranasally inoculated with different two doses [1 × 10^5^ for **(A**, **B)** and 1 × 10^7^ CFU/mouse for **(C, D)**] of *S. pneumoniae* without anesthesia on day 0. Nasal lavage and blood were collected on day 2 for counting colonies. Each symbol represents individual data. For low-dose groups **(A, B)**, 5 of TRPV1 KO, 5 of TRPV4 KO, and 5 of wild-type mice were used. For high-dose groups **(C, D)**, 14 of TRPV1 KO, 20 of TRPV4 KO, and 20 of wild-type mice were used. **(A, C)** Nasal lavage and **(B, D)** blood. Horizontal lines indicate median values. The detection limit (the dotted line) is 666 CFU/ml. **p < 0.01.

To evaluate the role of TRPV1 and TRPV4 for pneumococcal carriage, mice (wild-type, TRPV1 KO, TRPV4 KO) were intranasally inoculated with two different doses (1 × 10^5^ and 1 × 10^7^ CFU/mouse) of *S. pneumoniae* 6A strain without anesthesia (**Figure 1**). *S. pneumoniae* was successful in establishing nasal colonization at the same level in all mouse strains (**Figures 2A, C**). The density of colonization was dose-dependently increased among all mouse strains. At the same timing as nasal lavages, the blood cultures were also evaluated for detection of bacteremia. In the low-dose group, bacteremia was not detected in any mouse strains (**Figure 2B**). On the other hand, a significantly higher ratio of bacteremia was observed in TRPV4 KO mice (15 out of 31 mice, 48.4%) compared with wild-type (3 out of 20 mice, 15%) and with TRPV1 KO mice (4 out of 14 mice, 28.6%) by high-dose inoculation (**Table 1**). The density of bacteremia in TRPV4 mice was significantly higher than that of wild type by Kruskal–Wallis test (*p* = 0.028) (**Figure 2D**).

**Table 1:** Summary of incidence of bacteremia.

	Bacteremia (ratio)	*p* value *vs*. wild type by Fisher’s exact test
**Mono-infection** **High dose**		
Wild type	3/20 (15%)	–
TRPV1 KO	4/14 (28.6%)	Not significant (Ns)
TRPV4 KO	15/31 (48.4%)	*p* < 0.05
**Coinfection** **Low dose**		
Wild-type	0/12 (0%)	*-*
TRPV1 KO	2/9 (22.2%)	Ns
TRPV4 KO	4/9 (44.4%)	*p* < 0.05
**High dose**		
Wild-type	7/15 (46.7%)	–
TRPV1 KO	8/19 (42.1%)	Ns
TRPV4 KO	11/17 (64.7%)	Ns

**Figure 3:**
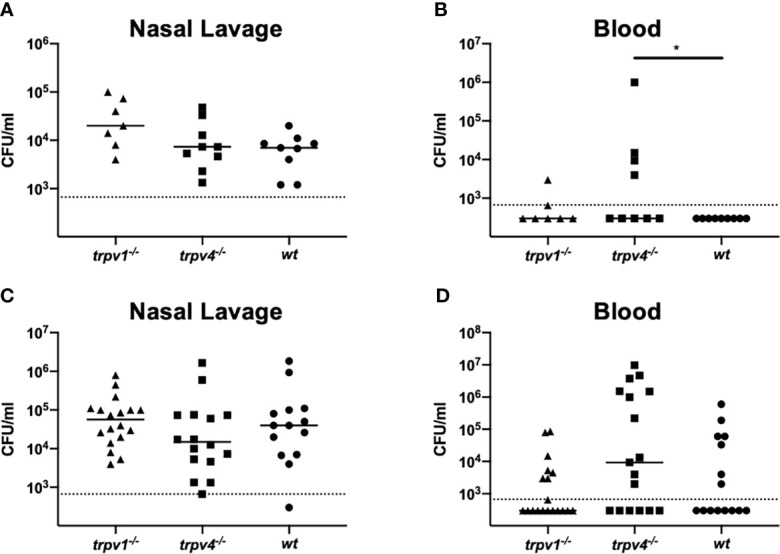
Nasal colonization and bacteremia in the coinfection model. After inoculation of IAV on day –2, mice were intranasally inoculated with two different doses [1 × 10^5^ CFU/mouse for **(A, B)** and 1 × 10^7^ CFU/mouse for **(C, D)**] of *S. pneumoniae* without anesthesia on day 0. Nasal lavage and blood were collected on day 2 for counting colonies. Each symbol represents individual data. For low-dose groups **(A, B)**, 7 of TRPV1 KO, 9 of TRPV4 KO, and 9 of wild-type mice were used. For high-dose groups **(C, D)**, 19 of TRPV1 KO, 17 of TRPV4 KO, and 15 of wild-type mice were used. **(A, C)** Nasal lavage and **(B, D)** blood. Horizontal lines indicate median values. The detection limit (dotted line) is 666 CFU/ml. *p < 0.05.

Next, we evaluated the impact of influenza virus coinfection, one of the most exacerbating factors of pneumococcal diseases, on nasal colonization and bacteremia. Even when the mice had been infected with influenza A virus (IAV), the density of nasal colonization was almost equal among all mouse strains, regardless of the dose of inoculum (**Figures 3A, C**). Unlike the pneumococcal mono-infection model, bacteremia was observed, even with the low-dose inoculum. Both the density and the incidence of bacteremia were significantly higher in TRPV4 mice than in wild-type mice (*p* = 0.036) (**Figure 3B**). Although bacteremia was detected at the same frequency in all mouse strains when the dose of inoculum was increased (**Table 1**), TRPV4 KO mice showed the highest density of bacteremia (**Figure 3D**). In this mouse model using a virulent pneumococcal strain, TRPV4 KO mice were more susceptible to development of bacteremia, although all mouse strains were colonized equally.

### Local Inflammatory Responses in the Nasal Cavity After the Pneumococcal Inoculum

**Figure 4:**
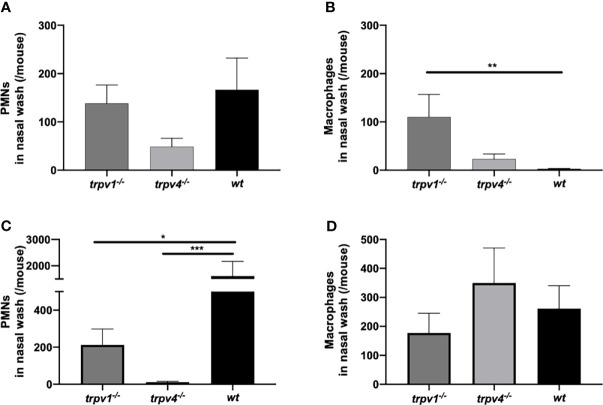
The number of PMNs and macrophages in the nasal lavages. The nasal lavages were collected on day 2 from the mice in the mono-infection and coinfection model. The numbers of PMNs and macrophages were counted by flow cytometry. **(A, B)** The mono-infection model and **(C, D)** the coinfection model. **(A, C)** the number of PMNs and **(B, D)** the number of macrophages. The mean value with the standard error of the mean was represented by a line bar. For the mono-infection model **(A, B)**, 15 of TRPV1 KO, 10 of TRPV4 KO, and 10 of wild-type mice were used. For the coinfection model **(C, D)**, 11 of TRPV1 KO, 12 of TRPV4 KO, and 10 of wild-type mice were used. *p < 0.05, **p < 0.01, and ***p < 0.001.

The local status of inflammation was analyzed with lavages of the upper respiratory tract. The number of PMNs in the nasal lavages on day 2 was counted by flow cytometry. The influx of PMNs was suppressed in TRPV4 KO mice compared to TRPV1 KO and wild-type mice (**Figures 4A, C**). Especially in the coinfection group, the number of PMNs was significantly lower in TRPV1 KO (*p* = 0.043) and TRPV4 KO (*p* = 0.0002) than that of wild type. Among TRPV4 KO mice, the number of PMNs was significantly lower in the coinfection model than in the mono-infection model (*p* = 0.018 by Mann–Whitney’s U test), which was the opposite result of an increase of PMNs in the coinfection model among wild-type mice.

We also counted the number of macrophages in the nasal lavages. There was a significant difference between TRPV1 KO and wild type in the mono-infection model (**Figure 4B**). In the coinfection model, there were no differences in the number of macrophages among the three mouse strains (**Figure 4D**).

### Dissemination of the Pneumococcal Infection to the Other Organs

**Figure 5:**
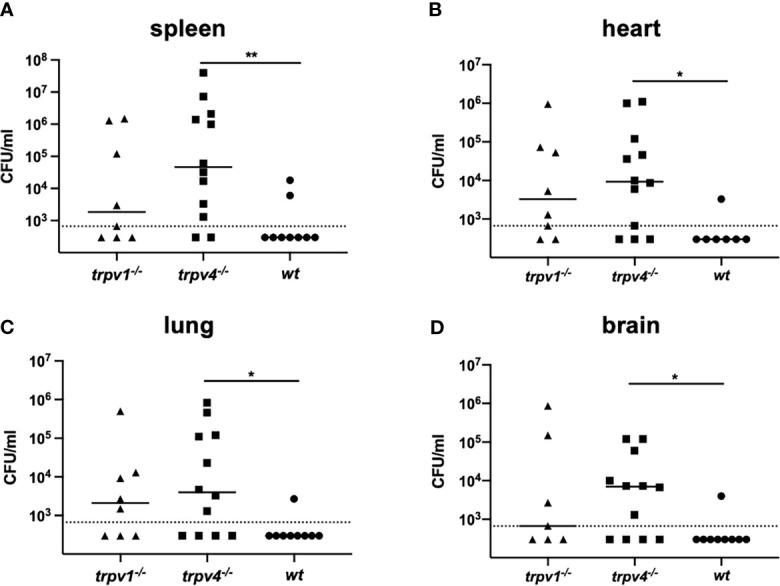
The number of pneumococcal colonies detected in various organs. The spleen **(A)**, heart **(B)**, lung **(C)**, and brain **(D)** were collected on day 3 from the mice in the mono-infection and coinfection models. The data of the coinfection model were displayed. Each sample was thoroughly washed and homogenized in 1 ml of sterile PBS. Each symbol represents individual data. Eight of TRPV1 KO, 12 of TRPV4 KO, and 7 of wild-type mice were used. Horizontal lines indicate median values. The detection limit (the dotted line) is 666 CFU/ml. *p < 0.05 and **p < 0.01.

To see the spread of the pneumococcus to various organs after nasal colonization, the mice were sacrificed on day 3 and the hearts, spleens, lungs, and brains were collected for culture (**Figure 1**). In the pneumococcal mono-infection model, we could not find bacteria in any organs in any mouse strains (data not shown). Contrary to wild type, the bacteria were detected in most of the samples of TRPV1 KO and TRPV4 KO mice in the coinfection model (**Figure 5**). Especially in the TRPV4 KO mice, the density of bacteria in all organs examined was significantly increased as compared with the wild type.

**Figure 6:**
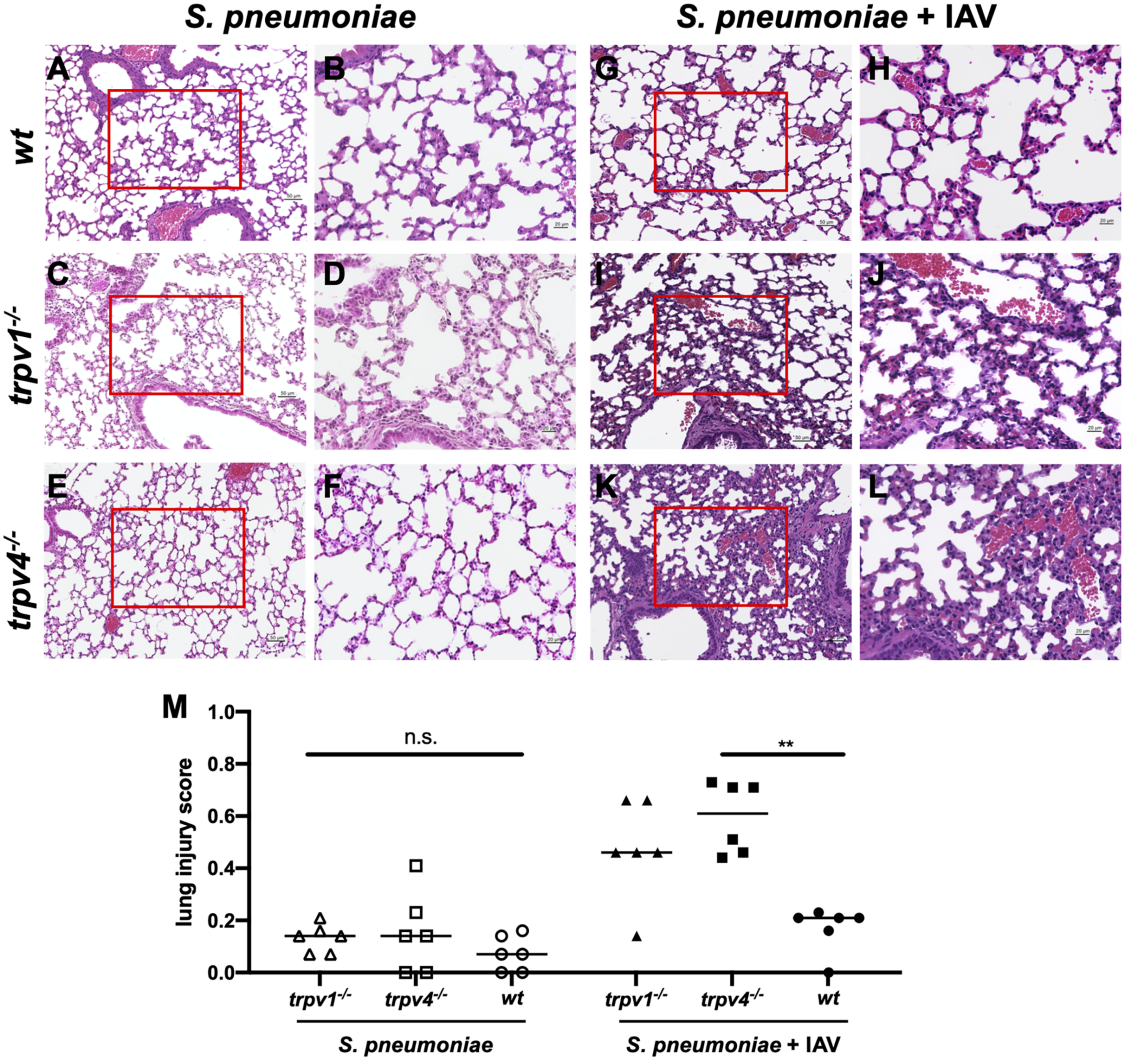
The histology of the lung. The H&E staining of the lung was performed with three mice for each group. The representative images were displayed in this figure. **(A–F)** The mono-infection model. **(G, L)** The coinfection model. **(A, B, G, H)** Wild type **(C, D, I, J)**, TRPV1 KO, and **(E, F, K, L)** TRPV4 KO. **(A, C, E, G, I, K)** Low magnification (×200. Scale bar, 50 µm) and **(B, D, F, H, J, L)** high magnification of boxed sections of the low-magnification field (×400. Scale bar, 20 µm). **(M)** Lung injury score. The severity of lung inflammation was quantified using the scoring system. Each symbol represents individual data. Horizontal lines indicate median values. (N = 6 for each group). n.s., not significant, **p < 0.01.

To investigate the route of pneumococcal infection in the multiple organs, the lung tissues were histologically analyzed. In the mono-infection model, no inflammation was observed in the lungs among all mouse strains (**Figures 6A**–**F**). In the coinfection model, the lungs of TRPV4 KO mice showed inflammatory infiltrates with neutrophils and macrophages in the interstitium and hemorrhage in the alveoli (**Figures 6K, L**). Furthermore, the lung tissues of TRPV1 KO mice demonstrated alveolar walls showing capillary congestion and mild to moderate inflammatory infiltrate including inflammatory cells (**Figures 6I, J**). On the other hand, no evidence of apparent inflammation was observed in the lung tissues of wild-type mice (**Figures 6G, H**). The severity of inflammation was quantified using a previously reported scoring system (24). There was a significantly higher inflammation in TRPV4 KO mice than wildtype when the mice were infected with both S. pneumoniae and IAV (**Figure 6M**). We also evaluated the lung tissues of the mice infected only with IAV and confirmed no apparent findings of viral pneumonia (data not shown). Histological findings suggest that TRPV1 and TRPV4 may be involved in regulating the pneumococcus migration to the lower respiratory tract and dissemination to multiple organs.

### Effect of TRPV on Pneumococcal Lethal Infection

**Figure 7:**
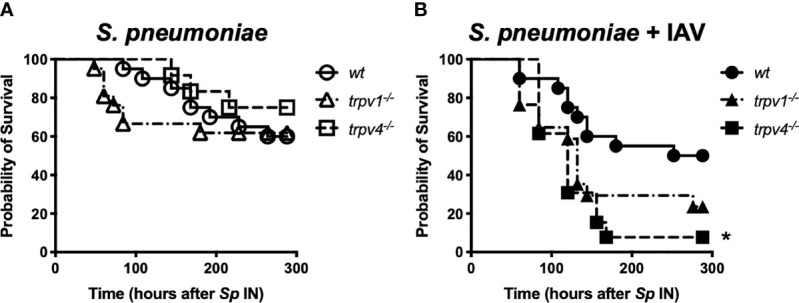
The time course of development of lethal infection. After intranasal administration of *S. pneumoniae*, the mice were monitored every 12 h until day 12. When the mice were found to be sick (decreased motor activity, shivering, and messy hair), they were immediately euthanized and the blood was collected for confirmation of bacteremia. **(A)** The mono-infection model and **(B)** the coinfection model. Circle, triangle, and square indicate wild-type, TRPV1 KO, and TRPV4 KO mice, respectively. *p < 0.05.

To evaluate the role of TRPV1 and TRPV4 on pneumococcal invasive diseases, we established a spontaneous pneumococcal lethal infection model after nasal colonization. The mice were given the bacteria intranasally on day 0 of the experiment with the dose of 1 × 10^7^/mouse. They were monitored every 12 h until they showed signs of lethal infection, such as decreased motor activity, shivering, and messy hair (**Figure 1**). Similar time courses were observed, and around 70% of mice finally developed lethal infection among all groups in the mono-infection model (wild type; 65%, TRPV1 KO; 62%, TRPV4 KO; 75%, respectively) (**Figure 7A**).

When the mice were pre-infected with IAV, more than 70% of TRPV1 KO mice eventually developed lethal infection. TRPV4 KO mice showed the worst course in the coinfection model, and all mice developed lethal infection within 7 days after the pneumococcal challenge. Wild type showed significantly better survival (50%) than TRPV4 KO mice (**Figure 7B**). We also observed the mice infected with only IAV, and none of the mice in any of the three strains died during the observation period (data not shown).

### Systemic Inflammatory Responses After the Pneumococcal Infection

**Figure 8:**
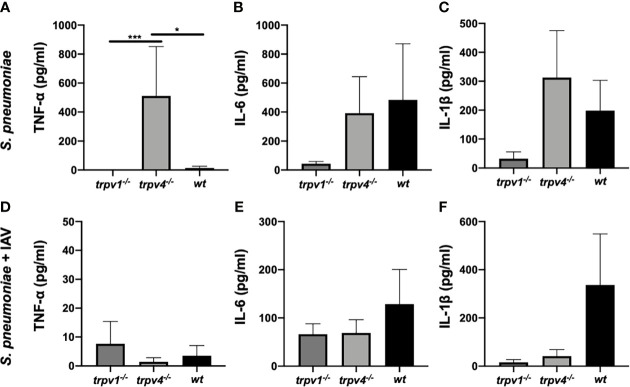
The levels of cytokines in the sera. The levels of cytokines in the sera were measured on day 2 of the experiment. The data were displayed as mean with standard error of the mean. **(A, D)** TNF-α, **(B, E)** IL-6, and **(C, F)** IL-1β. **(A–C)** The mono- infection model and **(D–F)** the coinfection model. For the mono-infection model **(A–C)**, nine of TRPV1 KO, eight of TRPV4 KO, and eight of wild-type mice were used. For the coinfection model **(D–F)**, 13 of TRPV1 KO, 11 of TRPV4 KO, and 8 of wild-type mice were used. *p < 0.05, ***p < 0.001.

Blood samples were collected from the colonized mice on day 2 of the experiment. The sera were separated by centrifugation, and the levels of cytokines—tumor necrosis factor-α (TNF-α), interleukin-6 (IL-6), and interleukin-1β (IL-1β)—were measured by ELISA. In the mono-infection model, the levels of TNF-α were significantly higher in TRPV4 KO mice than those of wild type and TRPV1 KO mice (*p* < 0.05 and *p* < 0.001, respectively) (**Figure 8A**). Similar levels of IL-6 and IL-1β were observed among wild-type and TRPV4 KO mice, whereas the production of IL-6 and IL-1β was low in TRPV1 KO mice (**Figures 8B, C**).

When coinfected with IAV, the production of TNF-α was greatly suppressed in TRPV4 KO mice compared with the mono-infection model (**Figure 8D**). The levels of IL-6 and IL-1β were lower in TRPV1 KO and TRPV4 KO mice than in wild-type mice, although without statistical differences (**Figures 8E, F**).

### Effect of TRPV1 and TRPV4 on the Phagocytic Bactericidal Ability of Neutrophils

**Figure 9:**
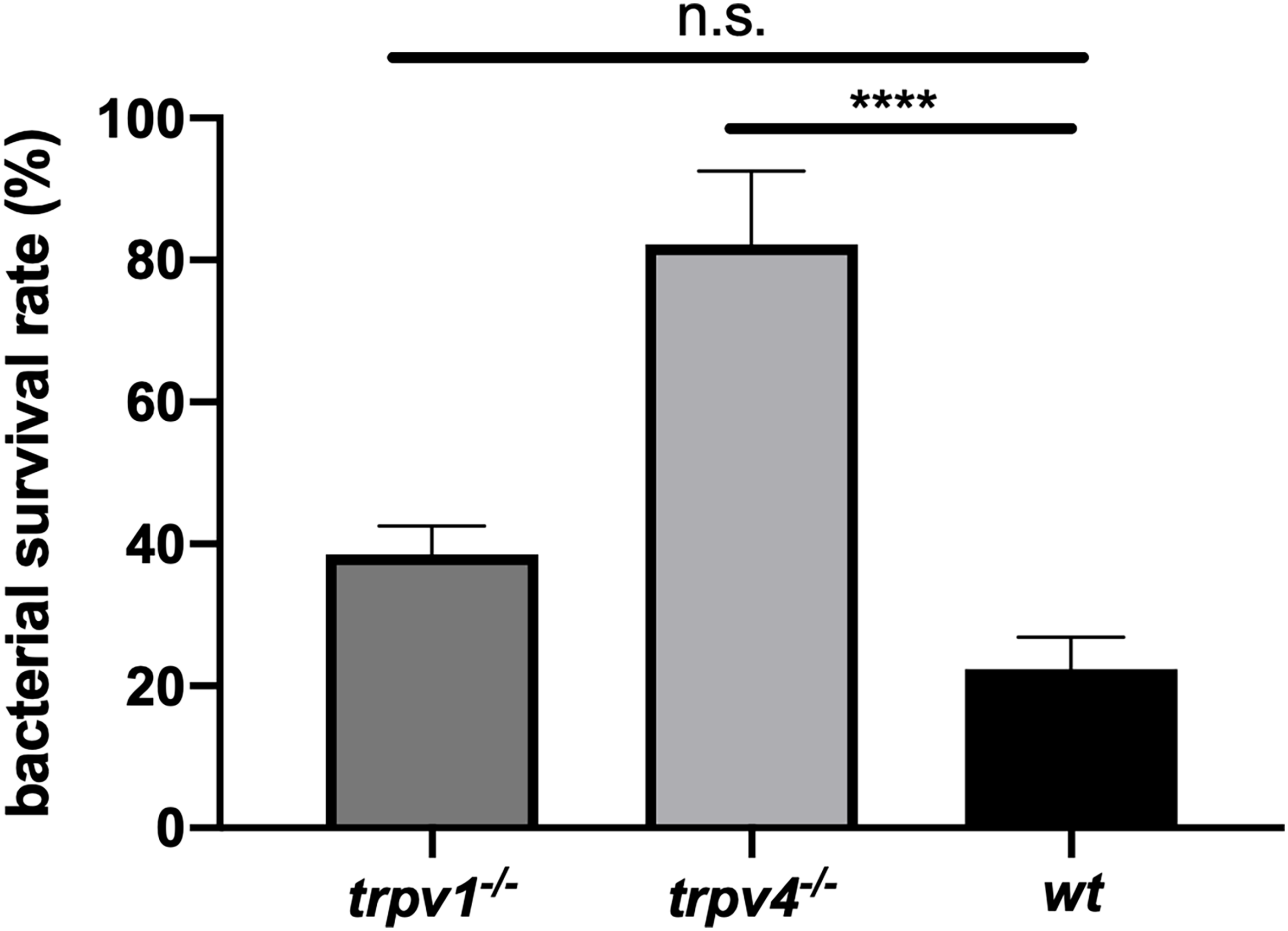
The phagocytic bactericidal ability of PMNs. PMNs isolated from each mouse strain were incubated with the bacteria (10^2^ CFU) pre-opsonized with baby rabbit complement for 45 min in a bacterium: PMN ratio of 1:1000. The bacterial survival rate was calculated by dividing the number of bacteria in the experimental sample by those in the control sample that did not contain PMNs. The assay was triplicated and repeated three times for each mouse strain. Pooled data were displayed (N = 9 for each group). The mean value with the standard error of the mean was represented by a line bar. n.s., not significant, ****p < 0.0001.

To evaluate the mechanism of protection against pneumococcal infection, we focused on the phagocytic bactericidal ability of neutrophils, which is one of the most effective systems for eliminating the pneumococcus. Neutrophils were isolated from each mouse strain and incubated with pre-opsonized bacteria with baby rabbit complement. The survival rate of the bacteria was compared between three groups. The experiment was repeated three times for each mouse strain, and the pooled data are displayed in **Figure 9**. Neutrophils derived from TRPV4 KO mice showed significantly lower bactericidal ability than those derived from wild type (*p* < 0.0001). TRPV1 KO mice showed a similar tendency, but no significant difference was observed.

## Discussion

In this report, we demonstrated for the first time that TRPV1 and TRPV4 were involved in the development of invasive pneumococcal infections after establishment of nasal colonization by using knockout mouse strains. Regarding nasal colonization, the pneumococcus could establish stable colonization at the same level among all three mouse strains with or without influenza virus coinfection. In previous studies, the role of TRP channels in infectious diseases has focused on a single-organ infection or a systemic infection (4–6), suggesting that TRP channels do not function positively in a carrier state that does not induce strong inflammation by *S. pneumoniae*.

On the other hand, knockout of TRPV channels showed a clear change in the development of pneumococcal infections. In the mono-infection model, the incidence of bacteremia significantly increased among TRPV4 KO mice by high-dose challenge. Even by low-dose challenge, TRPV4 KO mice frequently developed bacteremia when the mice were precedingly infected with influenza virus. It is well known that influenza infection induces strong inflammation with neutrophil influx in the respiratory tract; however, the number of neutrophils in the nasal lavages from TRPV KO mice was significantly low, suggesting the possibility of defect of neutrophil recruitment during influenza infection without TRPV channels. Recent reports have shown that TRP channels are expressed in various cells including immune cells and protect lung infection of Gram-negative bacteria *via* MAPK activation switching (6). We further investigated the bactericidal activity of neutrophils isolated from TRPV KO mice because phagocytosis of opsonized bacteria by neutrophil is one of the most important mechanisms for eliminating *S. pneumoniae*. Significantly impaired bactericidal function of neutrophils derived from both TRPV KO mice (especially TRPV4) ex vivo was observed, suggesting that the TRPV4 channel expressed on neutrophils would be one of the important factors that recognize the pneumococcus. Decreased local influx and phagocytosis of neutrophils might promote the migration of the pneumococcus to the lung and the development of invasion to the bloodstream. Little is known about the function of the TRPV4 channel during influenza infection, but these results suggest that TRPV4 plays a key role for preventing the exacerbation of pneumococcal infections. In this study, the mechanism of decreased recruitment and impaired bactericidal function of neutrophils lacking TRPV4 are unrevealed. Future studies should be focused on investigating the interaction between TRP channels and pneumococcal virulence factors such as pneumolysin.

In TRPV KO mice (especially TRPV4 KO), bacterial dissemination was observed in multiple organs 3 days after pneumococcal infection, although it was unlikely among wild type. Together with the histological findings of the lungs, most of TRPV KO mice were in pre-sepsis state *via* pneumonia on day 3 after pneumococcal infection resulting in worse prognosis than the wild type. Because the strain used in this study was virulent, even wild-type mice eventually developed lethal infection at a similar rate, as previously reported using the same background strain (23).

The current results are consistent with the other studies related to TRPV4 and its role in the lungs, where the TRPV4 channel is important in the mediation of the phagocytosis, regulation of cytokines, and generation of reactive oxygen species (ROS). The TRPV4 channel may protect from bacterial pneumonia caused by *Pseudomonas aeruginosa* through mitogen-activated protein kinase (MAPK) switching (6). The TRPV4 channel has been shown to be expressed in macrophages of the lungs, and it may have a role in the activation by mechanical stress (26). Another mechanism has been demonstrated that the E3 ubiquitin ligase TRIM29 regulated the activation of alveolar macrophages and had a critical role in the protection against LPS-induced sepsis by *Haemophilus influenzae* (27). Macrophage is also thought to be critical for the clearance of *S. pneumoniae* (28, 29). Flow cytometry showed a similar number of macrophages among all three strains in the coinfection model, although the pneumococcus was detected in multiple organs among only TRPV KO mice. This discrepancy suggests that elimination of *S. pneumoniae* by macrophages may require a TRP-mediated cognitive mechanism, and the timing of evaluating macrophage recruitment was before the peak (30). Scheraga et al. reported that TRPV4 distributed the phenotypic change of macrophage that resulted in the promotion of phagocytosis and bacterial clearance (31).

Regarding the evaluation of cytokines, a significant elevation of TNF-α among TRPV4 KO mice during pneumococcal mono-infection is a good reflection of the relationship between TRPV4 and inflammatory mediators. Xu et al. reported an administration of the TRPV4 agonist inhibited TNF-α-induced monocyte and leukocyte adhesion to human epithelial cells, resulting in reduced atherosclerotic plaque formation in a mouse model (32). The stimulation of LPS to cultured rat microglia expressing TRPV4 controlled the release of TNF-α (33), suggesting that bacterial virulence factors can directly activate TRP channels. Mice lacking the TRPV4 channel could not suppress the production of TNF-α after the blood invasion of *S. pneumoniae*. Contrary to the mono-infection model, the level of TNF-α in the sera of TRPV4 KO mice was very low in the coinfection model. The relationship between influenza virus and TRPV4 channel has not been reported as far as we are aware. During a viral infection that causes strong inflammation in the respiratory tract, TRP channels may respond more strongly and differently, so further investigations are needed. The limitation of the cytokine assay in this study is that the evaluation timing was fixed on day 2 because the mice started developing lethal infection 48 h after the pneumococcal challenge. There should be mice in various conditions such as pneumonia, bacteremia, and sepsis, resulting in a huge variability of cytokine levels within each group.

The serotype 6A pneumococcal strain we used in this study usually develops bacteremia by direct invasion from nasal colonization (without pneumonia) (18). Mice lacking TRPV1 or TRPV4 channels, however, showed moderate to severe pneumonia 48 h after the super-infection of the pneumococcus. This finding is similar to secondary pneumococcal pneumonia, which sometimes occurs as a fatal complication during an influenza pandemic (14). TRPV1 and TRPV4 channels are suggested to contribute to the suppression of pneumococcal pneumonia in the coinfection model. The detailed mechanism of how TRPV channels protected the development pneumonia or lethal infection was not evaluated in the current study. We firstly aimed to investigate the role of TRPV channels on the development of invasive pneumococcal infections following nasal colonization, which the pneumococcus naturally acts within the host. To reveal the detailed mechanism of TRPV channels on each pneumococcal disease such as pneumonia or septicemia, a direct administration to the targeted organ (intra-tracheal injection or intravenous/intraperitoneal injection) should be conducted in the future study separately.

Although no significant difference in survival rate was observed in the lethal infection experiment, the pneumococcus was successful in disseminating to various organs by coinfection of influenza virus, and moderate pneumonia was found among TRPV1 KO mice. Taylor et al. reported that the influenza virus-infected lower respiratory tract inhibited capsaicin and substance P induced relaxation responses (34). The block of the TRPV1 channel may lead the loss of bronchoprotective response during viral infection, which results in exacerbation of pneumococcal super-infection.

The history is relatively short because the link between TRPV channels and infectious diseases remains controversial. Especially for TRPV1, there are a couple of reports concluding that inhibition of TRPV1 neurons during bacterial infection results in a better prognosis. A study of lung infection caused by *Staphylococcus aureus* showed that ablating the TRPV1 nociceptors increased the lung bacterial clearing rate, induced cytokines, and enhanced survival by suppression of the neutrophil activity and by mediation of immunosuppression due to the release of calcitonin gene-related peptide (CGRP) (4). Another study in the *Streptococcus pyogenes* infections demonstrated the role of TRPV1 in the clearance of the pathogen as TRPV1 neurons could be activated by streptolysin S and promoted by the release of CGRP in the infected tissues, therefore increasing the lethality by blocking the recruitment of neutrophils (5). To explain the discrepancy of the current result in TRPV1 KO mice, future studies will investigate how TRP channels recognize the pneumococcal virulence factors and function positively or negatively within the host immune system.

In conclusion, both TRPV1 and TRPV4 channels regulated the development of pneumococcal infections arising from nasal colonization in an adult mouse model. The TRPV4 channel in particular may play an important role in the prevention of secondary pneumococcal pneumonia and its subsequent development of lethal infection during influenza virus infection. Regulating host immune responses with TRP channels would be a novel strategy against various pathogenic microorganisms.

## Data Availability Statement

The original contributions presented in the study are included in the article/**Supplementary Material**. Further inquiries can be directed to the corresponding author.

## Ethics Statement

The animal study was reviewed and approved by the Institutional Animal Care and Use Committee at Wakayama Medical University.

## Author Contributions

MK, SS, and MHo conceived and designed the experiments. DN, MK, HS, DM, MHi, TK, TS, FK, KE, and MM performed the experiments. DN, MK, HS, DM, and GS analyzed the data. MK, DN, and MHo wrote the paper. YO and SS revised the manuscript. All authors contributed to the article and approved the submitted version.

## Funding

This project was supported by JSPS KAKENHI Grant Number 19K08959 to GS and JSPS KAKENHI Grant Number 20K08825 to MK.

## Conflict of Interest

The authors declare that the research was conducted in the absence of any commercial or financial relationships that could be construed as a potential conflict of interest.

## Publisher’s Note

All claims expressed in this article are solely those of the authors and do not necessarily represent those of their affiliated organizations, or those of the publisher, the editors and the reviewers. Any product that may be evaluated in this article, or claim that may be made by its manufacturer, is not guaranteed or endorsed by the publisher.
