# Supplementary material for: The Roles of Transient Receptor Potential Vanilloid 1 and 4 in Pneumococcal Nasal Colonization and Subsequent Development of Invasive Disease
Source: Front Immunol. 2021 Nov 3;12:732029. doi: 10.3389/fimmu.2021.732029 (PMC8595402; doi:10.3389/fimmu.2021.732029)
Supplement: Supplementary file 1 [file Table_1.docx]

**Supplementary Table. Lung Injury Scoring System**

|  | Score per field | | |
| --- | --- | --- | --- |
| Parameter | 0 | 1 | 2 |
| A. Neutrophils in the alveolar space | none | 1-5 | >5 |
| B. Neutrophils in the interstitial space | none | 1-5 | >5 |
| C. Hyaline membranes | none | 1 | >1 |
| D. Proteinaceous debris filling in the airspaces | none | 1 | >1 |
| E. Alveolar septal thickening | <2x | 2x-4x | >4x |

Score = ((20 x A) + (14 x B) + (7 x C) + (7 x D) + (2 + E)) / (number of fields x 100)
